# Stated Preference Research in Otolaryngology: A Scoping Review

**DOI:** 10.1002/oto2.70140

**Published:** 2025-06-12

**Authors:** Lucy Xu, Molly N. Huston, Victoria S. Lee, John D. Cramer, Deborah Goss, Matthew R. Naunheim

**Affiliations:** ^1^ Harvard Medical School Boston Massachusetts USA; ^2^ Department of Otolaryngology–Head and Neck Surgery Massachusetts Eye and Ear Boston Massachusetts USA; ^3^ Department of Otolaryngology–Head and Neck Surgery Washington University in St. Louis St. Louis Missouri USA; ^4^ Department of Otolaryngology University of Illinois at Chicago Chicago Illinois USA; ^5^ Department of Otolaryngology Wayne State University School of Medicine Detroit Michigan USA

**Keywords:** best‐worst scaling, contingent valuation, discrete choice experiment, otolaryngology, patient preference, scoping review, willingness‐to‐pay

## Abstract

**Objective:**

Stated preference research methods, including discrete choice experiments (DCEs), conjoint analysis (CA), best‐worst scaling (BWS), and willingness‐to‐pay/contingent valuation (WTP/CV) studies, are excellent tools for understanding patient preferences in healthcare. Their application in otolaryngology has yet to be described. This work encompasses a scoping review assessing the field of stated preference research in otolaryngology, to identify gaps in the current literature and identify areas of future applications of such methodologies.

**Data Sources:**

Embase, Medline, and Web of Science.

**Review Methods:**

A search of three databases for all relevant publications through 2023 was performed using relevant search terms. Eligibility criteria for included studies included the use of one of four methodologies (DCE, CA, BWS, and WTA/CV). After screening and full‐text review by two authors, data were extracted, including relevant methodologic parameters including type of study, survey development characteristics, sample size, and outcome. Data were analyzed using descriptive statistics.

**Results:**

Of 3064 search results, 57 were included for full data extraction from inception to 2023, across 14 countries, with an increasing number of studies in recent years. WTP/CV was the most common method (58%), followed by DCE (30%), CA (23%), and BWS studies (5%). Otology was the most frequently studied subspeciality (36.8%). Treatment options were more commonly studied than diagnostics or health state preferences. Many studies did not specify survey development methods (38.6%).

**Conclusion:**

Stated preference research in otolaryngology is relatively sparse, and there is significant methodological inconsistency in the development and implementation of these methods. This review provides research priorities for stated preference research in otolaryngology in an era of patient‐centered care.

**Level of Evidence:**

Level 4.

Otolaryngologists strongly value the input of their patients when making treatment decisions. Patients likewise value the input of their physicians and must integrate their preferences with this expert opinion about risk and benefit to make choices about their medical care. In otolaryngology, many conditions are “preference sensitive” insofar as there are multiple acceptable treatment options, without a clear superior choice based solely on medical and scientific research.^1^, ^2^ An example of such a condition is an early‐stage laryngeal cancer. Although both radiation and surgery may be good options for the patient, there is relative equipoise between these options in many clinical scenarios, and both treatments can be offered to the patient. Because otolaryngology has a high proportion of elective and quality of life interventions, the preferences of the patient, informed by their personal beliefs and their physician's counsel, are of paramount importance.

To date, rigorous evaluation of patient preferences for assessment or treatment of otolaryngologic conditions has not been integrated into clinical practice or research. Indeed, there is little awareness of the formal stated preference elicitation methods in otolaryngology.^3^ The objective of the present work is to assess the current state of stated preference research in otolaryngology. As there is a dearth of high‐quality studies in this area, a scoping review (as opposed to a systematic review) was chosen as an exploratory, rather than explanatory, inquiry into the existing literature. In particular, we seek to identify the types of preference studies being used to study otolaryngologic conditions, the process by which these methodologies are implemented, the populations (adult vs pediatric; diseased vs general population) studied, and the fields within otolaryngology that have utilized this research paradigm the most. We hypothesize that there are significant gaps in the methodological approaches to patient preference research within otolaryngology.

There are several key subtypes of preference studies considered in this scoping review. Although there are many ways to elicit preferences from patients, including everything from qualitative and open‐ended questions to quantitative choice questions, we herein focus on the most commonly used tools.^4^, ^5^, ^6^ Choice‐based tasks, like discrete choice experiments (DCEs) and conjoint analysis (CA), function based on the premise that patients decide between options based on the underlying attributes of those options, making trade‐offs between potential risks and benefits. By constructing a research scenario in which patients are asked to select among carefully constructed choice sets, the underlying preferences for those attributes of care or treatment are elucidated in a quantitative, reproducible fashion that provides both individual and population‐level preferences.^2^ Although the terms CA and DCE are often used interchangeably, there are differences in their theoretical underpinnings, and they achieve different research objectives.^7^ Ranking methods, most notably best‐worst scaling (BWS), ask participants to rank the best and worst options among several choices. These methods are superior to standard rating scales (eg, “please rate the importance of X on a scale of 0 to 10”) insofar as they avoid issues with scale region bias, discrimination, and consistency.^8^ Contingent valuation (CV), also known as willingness‐to‐pay (WTP), is a threshold technique designed to measure the value of a medical product, good, service, or intervention from the patient's perspective using constructed hypothetical scenarios. DCE, CA, BWS, and CV/WTP were all included in this review. Of note, certain types of studies were excluded from this review, including time trade‐off, visual analog scale, and standard gamble techniques, as these have been explored elsewhere and are typically used for utility elicitation in the context of cost‐utility studies,^9^, ^10^, ^11^ as well as simple ranking/rating exercises.

## Methods

### Protocol and Registration

The Preferred Reporting Items for Systematic Reviews and Meta‐analyses Extension for Scoping Reviews (PRISMA‐ScR) was used in the planning of this study and the preparation of the manuscript. The protocol was thereafter registered with the Open Science Framework (https://osf.io/).

### Eligibility Criteria

Several criteria were utilized to frame this review. Broadly this included the following: (1) studies within the otolaryngology literature and referring to the treatment of otolaryngologic conditions; (2) a timeframe including any literature published up until December 2023; (3) studies that assessed patient preferences; and (4) utilization of patient preference methodologies including CV or WTP research, BWS methodology, DCEs, and CA. Of note, many DCEs include WTP estimation within choice sets; these were categorized as both DCE and WTP. Excluded studies included those that did not pertain to otolaryngology, those researching preferences not related to the patient (eg, provider preferences), those looking at revealed preference (vs stated preferences), cost‐utility studies, and utility elicitation studies. Although adjacent to stated preference research, cost‐utility and utility elicitation studies are distinct and have been extensively reviewed in the otolaryngology literature. Several topics were otolaryngology‐adjacent (eg, gastroesophageal reflux disease, oral‐maxillofacial conditions), and these studies were excluded unless explicitly related to the otolaryngologic manifestations of these conditions. Conversely, a study on thyroid surgery performed by a general surgery researcher would be included despite not surveying patients in an otolaryngology setting. Only English language studies were included. Conference proceedings and posters without accompanying manuscripts were excluded, as were reviews, opinion pieces, and letters to the editor.

### Information Sources and Search Strategy

We searched Embase, Medline, and Web of Science for all relevant publications through the index date through the end of 2023. This search was conducted on January 16, 2024, by a research librarian (D.G.). The search was structured around two main parameters: the identification of papers related to otolaryngology and the identification within this group of papers related to patient preference research (see Supplemental Figure S1, available online, for a full list of search terms).

### Selection of Sources and Evidence

Author, title, and abstract information were imported into Covidence (Veritas Health Innovation), a web‐based collaboration software platform that streamlines the production of systematic, scoping, and other literature reviews. Duplicates were removed. Screening was independently performed by two authors (L.X. and M.R.N.), and conflicts were resolved by consensus. Full texts were then retrieved and uploaded for review, and studies were assessed for inclusion. Additional studies in the reference lists of the included studies were added if eligible.

### Data Items, Charting, and Process

Data were extracted from the selected studies and compiled into a database using Microsoft Excel. The form was developed by two authors and designed to capture similar information to other systematic and scoping reviews in other health fields.^12^, ^13^, ^14^ Information extracted included author names, title, year, country of origin, objective/purpose of research, subspeciality within otolaryngology, type of preference study, population, type of preference measured, probability sampling data, sample size, methods for determination of attributes or WTP values, type of CV/WTP if applicable, and attribute/choice set information if applicable. Sample size was defined as the number of participants who actually participated, rather than those asked to participate. Subspecialty was interpreted inclusively; for example, a study on pediatric cochlear implantation would be categorized as both otology and pediatrics. Studies without a clear subspecialty were coded as general otolaryngology. Categorization of the type of study was interpreted inclusively; for example, a DCE that used a cost attribute for WTP was coded as both DCE and WTP. The open‐ended method of asking value questions for WTP studies was assumed to be the default when not explicitly identified by the authors. The use of probability sampling was defined as any sampling method that involved randomization of who received the survey, as opposed to randomization of choice blocks *within* the survey. All studies deemed eligible underwent an extraction process without exclusion of studies, as the goal of the scoping review was to assess all relevant literature, regardless of quality, rather than critical appraisal for determination of inclusion or exclusion.

### Synthesis of Results

The collected data were assessed according to several main avenues of inquiry. The primary assessment was the number and types of stated preferences studies performed in otolaryngology. Secondary assessment included a subspecialty, condition, and target population analysis, as well as an exploration of the specific methodology differences between studies (eg, sample size, methods of determination of attributes and levels, and use of probability sampling). Descriptive statistics were used. Given that multiple categories of data collection allowed for more than one answer, overall percentages for each category (eg, study types, preferences measured) were allowed to add to greater than 100%.

## Results

### Search Results

**Figure 1:**
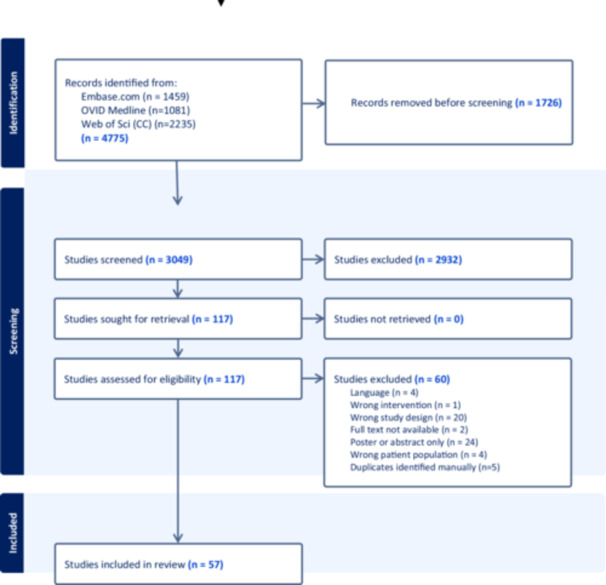
Preferred Reporting Items for Systematic Reviews and Meta‐analyses diagram.

The flow of studies through our scoping review is shown in the PRISMA diagram in Figure 1. Our search strategy supplied 3064 results, 57 of which were included for full data extraction. Supplemental Table S1, available online, summarizes the included studies categorized by type of state preference technique (DCE, BWS, CA, and WTP/CV; column 8, “Study Type”).

**Table 1:** Characteristics of Studies

Characteristic	n	%
Region		
North America	34	59.6
Europe	22	38.6
Asia	4	7.0
Australia	2	3.5
Time		
1990s	3	5.3
2000s	18	31.6
2010s	18	31.6
2020‐2023	18	31.6
Participants		
Adults with disease/problem	36	63.2
Adults from general population	13	22.8
Parents of children with disease/problem	9	15.8
Parents of children from general population	1	1.8
Study types		
DCE	17	29.8
CA	13	22.8
WTP/CV	33	57.9
BWS	3	5.3
Preference measured		
Health state	5	8.8
Diagnosis	3	5.3
Treatment (procedure)	25	43.9
Treatment (medication)	16	28.1
Treatment (device)	16	28.1
Subspecialty		
Rhinology	9	15.8
Otology/audiology	21	36.8
Laryngology	3	5.3
Pediatrics	6	10.5
Head and neck oncology	6	10.5
Facial plastics	5	8.8
Allergy	10	17.5
Sleep	3	5.3
General	7	12.3
Sample size		
<50	4	7.0
51‐100	9	15.8
101‐150	11	19.3
151‐200	8	14.0
>200	25	43.9
Development method		
Expert opinion	15	26.3
Literature review	21	36.8
Interview	16	28.1
Focus group	5	8.8
Pilot study	15	26.3
Other	4	7.0
Not specified	22	38.6

Abbreviations: BWS, best‐worst scaling; CA, conjoint analysis; CV, contingent valuation; DCE, discrete choice experiment; WTP, willingness‐to‐pay.

### Characteristics of Sources of Evidence

**Figure 2:**
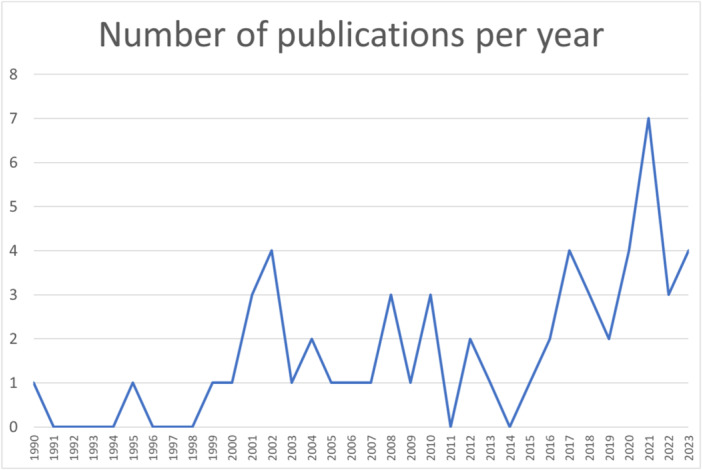
Run chart demonstrating the number of articles published by year.

Characteristics of the included studies are shown in Table 1. Sources included ranged from 1990 through 2023, with increasing frequency in the later time periods of the study (Figure 2); more than half of the studies occurred after 2016, with a peak number of 6 in 2021. Research was based in 14 countries (United States n = 27, Germany 9, Canada 7, United Kingdom 5, the Netherlands 4, Denmark 3, Australia 2, and 1 each from Spain, Denmark, Switzerland, Italy, India, Thailand, China, and Israel). Forty‐seven studies (82%) reported funding sources.

Among the subspecialties, otology/audiology had the greatest number of studies (37%), with approximately one in every five studies included focusing on hearing aids (19%). There were also many studies in allergy (18%) and rhinology (16%). All nine studies included under the rhinology designation focused on allergic rhinitis, and all of these focused on medication administration of nasal sprays, and most were funded by pharmaceutical companies or used branded medications.^15^, ^16^, ^17^, ^18^, ^19^, ^20^, ^21^, ^22^ Sleep (5%) and laryngology (5%) were the least well‐represented of the subspecialities.

The methods of development were varied among the 57 included studies, with 47% including more than one method to develop the patient preference assessment survey/tool. Methods of assessment that included patient input, including interviews, focus groups, and pilot studies, were used in only approximately half of the included studies (46%), whereas a significant proportion (12%) reported using only methods that are not patient‐derived, namely, expert opinion and literature review. A substantial minority (39%) of studies provided no explanation of how their preference tools were developed. Of this group, the majority (84%) focused on WTP/CV.

Of the study types investigated, WTP/CV was the most common type (58%), followed by DCE (30%), CA (23%), and relatively few BWS studies (5%). Most of the studies focused on adults, with 63% of patient preference surveys focusing on adults with the disease, and 23% focused on studies of the general population. Fewer studies focused on the parents or caregivers of children (18%). The plurality of studies was undertaken to understand preferences for surgeries and procedures (44%), with a substantial amount designed around understanding medications (28%) and devices (28%). Sample sizes varied widely from 30 to 2054, with a mean of 285 participants (median 171). Approximately 44% of studies had more than 200 participants. Only 13 studies engaged in probability sampling or any form of randomization within the population of interest (23%).

### WTP/CV

Thirty‐two studies focused on WTP/CV methodologies. The topics within this group were diverse, ranging from delivery of specialty care focused in emergency rooms^23^, ^24^ and through telehealth.^25^ A large number focused on otologic interventions for hearing loss, including hearing aids^26^, ^27^, ^28^, ^29^, ^30^ and cochlear implantation^31^, ^32^; devices for tinnitus relief were also explored.^33^, ^34^ Although the majority of studies focused on treatments, a few focused on valuing specific health states, such as the value of a night of restful sleep,^35^ the value of avoiding hearing loss,^36^ or a day of sore throat symptoms.^37^

Methodologically, 17 studies did not specify the detailed methodology of WTP/CV survey development.^15^, ^25^, ^29^, ^30^, ^32^, ^33^, ^34^, ^36^, ^37^, ^38^, ^39^, ^40^, ^41^, ^42^, ^43^, ^44^, ^45^ The rest used a combination of literature review, pilot studies, and expert opinion. The method of administration of the payment question was open‐ended in 38% of WTP studies,^15^, ^22^, ^27^, ^29^, ^30^, ^35^, ^36^, ^38^, ^40^, ^42^, ^46^ but payment cards,^16^, ^23^, ^24^, ^25^, ^27^, ^32^, ^33^, ^34^, ^37^ bidding games,^31^, ^43^, ^45^, ^46^ visual analog scale,^39^, ^41^, ^44^ and dichotomous choice^15^, ^23^, ^31^, ^47^ were also used, with several studies using multiple payment questions of varying methodologies.^15^, ^23^, ^27^, ^31^, ^46^ Finally, five publications included WTP as part of a larger DCE, using a cost or price variable among the attributes of the DCE.^26^, ^28^, ^48^, ^49^, ^50^

### DCEs/CA

There were 17 DCEs and 13 CAs. The CA group, the majority of studies (8 of 13) were within otology, whereas within the DCE group, there was a more even spread of subspecialities. Hearing aids and devices were commonly studied,^26^, ^28^, ^49^, ^51^, ^52^, ^53^, ^54^, ^55^, ^56^ as was cancer/tumor treatment.^57^, ^58^, ^59^, ^60^, ^61^, ^62^ There were several topics explored only by single publications, such as airway stenosis,^63^ facial paralysis,^64^ cosmetic concerns around thyroidectomy scars,^48^ and obstructive sleep apnea.^50^

Attributes numbered an average of 5.5 (median 5, range 3‐11), reflecting a balance of greater information and avoidance of survey burden. These attributes were most commonly determined via patient‐centric methods including pilot studies, interviews, and focus groups, but a small proportion did not record any justification for the tool development.^16^, ^17^, ^18^, ^25^, ^61^ Participants were given ample choice sets to register their preferences with an average of 13.3 (median 10, range 4‐64). DCEs tended to have the highest number of participants overall with the top 3 of all 57 preference studies all being DCEs with greater than 1000 participants.^16^, ^18^, ^57^

### BWS

Only three BWS studies were reported in head and neck, laryngology, and rhinology/allergy.^19^, ^65^, ^66^ These were all published within the past 10 years. Two focused on preferences for care delivery,^65^, ^66^ whereas the latter investigated preference for sensory attributes for nasal sprays.^19^ The number of attributes varied from 4 to 11, whereas all studies had a high number of choice sets.

## Discussion

In this scoping review of stated preference methods in otolaryngology, we identified 57 studies meeting criteria for inclusion, with all subspecialities represented. Most studies employed WTP/CV (58%) and DCE (30%) methodologies, with fewer using CA (23%) and BWS (5%). Whereas the former are well‐established research paradigms within healthcare, the paucity of BWS studies likely reflects the relatively recent emergence of BWS methodologies in healthcare.^67^, ^68^ Additionally, although CA and DCEs are related approaches with separate theoretical underpinnings,^7^ DCEs seem to have become more popular than CA in recent years with more CA in the 1990s and 2000s, and more DCE in the 2010s. Research was conducted in high‐income countries primarily, particularly the United States, Germany, and Canada, with studies increasing in frequency since 2017. At a high level, this review emphasizes the growing but still relatively limited use of stated preference research methodologies in the otolaryngology literature, and it highlights the potential of such research in healthcare decision‐making and personalized medicine.

The scope of topics encompassed in this review was broad, in both pediatric and adult patients, and with reference to each subspecialty. Nonetheless, certain topics have been more extensively studied, including hearing devices like hearing aids and cochlear implants, as well as allergic rhinitis. There are several potential reasons for the relatively high concentration in certain topics. First, this may be driven by specific authors with multiple publications in stated preference research within a particular niche, such as Grutters^27^, ^28^, ^56^ or Meister^51^, ^54^, ^55^ within the otology and audiology literature. Another potential reason for this relates to the nature of the lack of real‐world data that exists for specific topics. A majority of studies investigated the attributes of procedures, medications, and devices being developed, rather than diagnostics or health state assessment; indeed, stated preferences are particularly helpful for studying hypothetical scenarios or products that do not yet exist, to understand what consumers or patients may want.^69^ Furthermore, certain topics may be particularly sensitive to patient preferences and attitudes. Hearing aids and nasal sprays, for instance, require a high level of patient involvement in their daily use. The fit and sound emanating from the hearing aid likely determines its quotidian use; the feel and efficacy of a nasal spray impact adherence and WTP. In this way, certain “preference‐sensitive” conditions are much more likely to be studied than those that are not, such as head and neck trauma or the decision to remove a life‐threatening cancer.^2^ Finally, it cannot be overlooked that stated preference research, which is heavily utilized in consumer research, may serve to bolster economic decisions by companies designing consumer products. Its prevalence in certain niche areas may reflect market potential, as with the many studies related to rhinitis funded by pharmaceutical companies.^15^, ^16^, ^17^, ^18^, ^19^, ^20^, ^21^

Our review also highlights gaps in the literature. Certainly, otolaryngology suffers from a lack of robust research in patient preferences. Within HIV medicine, a small subset of the field of infectious disease, a recent review^13^ identified 57 stated preference studies for this one specific topic within infectious disease—the same number as identified in the entire field of otolaryngology. Randomization was also not routinely performed in the otolaryngology stated preference research literature. Most studies (77%) used convenience samples, which may predispose study results to bias and a lack of generalizability. It should be noted, however, that in some circumstances, such probability sampling is neither desired nor feasible, and it should be considered in the context of the study design and population of interest.

Additionally, the methodologies used to develop preference surveys demonstrate inconsistent adherence to best practices. Approximately 40% of studies did not identify how the surveys used to understand the opinions of participants were developed. Stated preference research should not only report this, but ideally should include patient perspectives during the development of the instruments in use.^70^ This includes qualitative interviews, focus groups, and pilot testing with patients or potential patients, and often requires an iterative process to ensure that appropriate attributes are being assessed.^70^ Such qualitative pretesting is a prerequisite for valid survey design, and it affects the accuracy and validity of study results.^69^, ^71^ Expert opinion and literature review alone, although expeditious, may be inadequate for many research questions. Dusseldorp et al provide an example of a well‐developed survey, which used iterative pilot testing, educational videos, and visual aids using data and survey responses from experts, patients, and the lay public regarding opinions about facial nerve paralysis.^64^ In contrast, another study attempting to explore barriers to the utilization of cochlear implants assesses preference without attempting to integrate patient input, thereby potentially excluding attributes of preference unexplored by clinicians.^32^

Several research priorities emerge from this work. First, the call for patient‐centered care and shared decision‐making that currently underpins healthcare reform depends on a deeper understanding of what patients think about, value, select, and pay for, and this review draws attention to the relative paucity of literature in this regard. Fields within otolaryngology, like the management of thyroid nodule and the care of patients suffering from head and neck cancer, are ripe for robust preference research.^44^, ^59^, ^72^, ^73^, ^74^ Expanding this beyond high‐income countries, from which most of the above literature originates, will be critical as the healthcare infrastructure develops in lower‐income countries. Researchers will find that several methodologies lend themselves to certain topics within otolaryngologists. In situations where a product or service is being developed for which there are many potential attributes, BWS may provide the most expedient (and easily understood) form of patient preference research. DCEs may be particularly helpful where specific modeling is required to predict patient choices, as these can provide quantifiable utilities for various attribute options. For researchers working with pediatric patients, distinct choice trade‐offs may be difficult, but qualitative patient preferences may be more easily assessed. Ultimately, the choice of stated method preference depends not only on the patient population but also on the type of output information required.

There are several limitations to this scoping review. Although we applied a systematic approach to review the topic of stated preference research in well‐defined categories, there are sources of information that are unexplored herein. Primarily, some research studies included in the initial review of search terms included ranking or qualitative methodologies that employed other techniques. We deliberately excluded these studies given the intention of understanding the field of stated preference methodology, which has clear theoretical underpinnings and best practices. Additionally, because of this focus on methodology, gray literature, defined here as information created and shared outside of typical academic publications, was not included. Finally, there is an inherent weakness in using preexisting literature to define the field, as there is likely significant publication bias; indeed, these are the gaps we intended to explore with this scoping review.

## Conclusions

Stated preference research in otolaryngology continues to be relatively sparse but appears to be increasing over time. Assessment of treatment preferences regarding surgeries, devices, and medication is more common than assessment of health states or diagnostics. Importantly, there is significant methodological inconsistency in the development and implementation of stated preference research, which highlights a priority for future research. This review serves to help clinicians, researchers, and policymakers interpret this scoping review in the context of the dire need for preference elicitation in an era of patient‐centered care.

## Author Contributions

**Lucy Xu**, contributed to the design of the work, served as a primary reviewer for articles, drafted the work, revised it critically for important intellectual content, provided final approval, accountability for work; **Molly N. Huston**, contributed to the concept and plan for the work, assessment of relevant information for the work, provided important intellectual content and final approval of the version to be published, accountability for work; **Victoria S. Lee**, contributed to the concept and plan for the work, assessment of relevant information for the work, provided important intellectual content and final approval of the version to be published, accountability for work; **John D. Cramer**, contributed to the concept and plan for the work, assessment of relevant information for the work, provided important intellectual content and final approval of the version to be published, accountability for work; **Deborah Goss**, conception of the work, bibliographic support and database management, drafting the work and revising it critically for important intellectual content, final approval of the version to be published, accountability for work; **Matthew R. Naunheim**, contributed to the design of the work, served as a primary reviewer for articles, drafted the work, revised it critically for important intellectual content, provided final approval, accountability for work.

## Disclosures

### Competing interests

None.

### Funding source

Dr. Naunheim is a consultant for Kytarro Limited, Mosanna Therapeutics, and Best‐in‐Class MD for work unrelated to this paper. This work was supported in part by the AAO‐HNS Cochrane Scholars Grant.

## Supporting information

Supplemental Figure S1. Search strategy. This document shows the search terms used in Embase, Medline, and Web of Science, encompassing terms relevant both to patient preference methods and otolaryngology. The number of resulting hits is also included.

Supplemental Table S1.
