# Supplementary material for: Stated Preference Research in Otolaryngology: A Scoping Review
Source: OTO Open. 2025 Jun 12;9(2):e70140. doi: 10.1002/oto2.70140 (PMC12160337; doi:10.1002/oto2.70140)
Supplement: Supplementary file 1 — Supplemental Figure S1. Search strategy. This document shows the search terms used in Embase, Medline, and Web of Science, encompassing terms relevant both to patient preference methods and otolaryngology. The number of resulting hits is also included. [file OTO2-9-e70140-s001.docx]

**Supplemental Figure 1.**

Some of the ORL terms were adapted from Peterson AM, Miller B, Ioerger P, et al. Most-cited patient-reported outcome measures within otolaryngology—revisiting the minimal clinically important difference: a review. JAMA Otolaryngol Head Neck Surg. Published online February 2, 2023. doi:10.1001/jamaoto.2022.4703

**Embase.com 1.16.2024**

('contingent valuation'/exp OR 'discrete choice experiment'/exp OR 'Willingness To Pay'/exp OR 'best worst scaling'/exp OR 'willingness to accept'/exp) OR ('stated preference*' OR 'conjoint analysis' OR 'conjoint survey' OR 'choice model' OR 'discrete choice' OR DCE OR 'best-worst scaling' OR 'willingness to pay' OR WTP OR 'willingness to accept' OR 'contingent valuation'):ti,ab,kw

**34,051**

'ear nose throat disease'/exp OR 'otorhinolaryngology'/exp OR 'nose disease'/exp OR 'epistaxis'/exp OR 'larynx disorder'/exp OR 'sinusitis'/exp OR 'smelling disorder'/exp OR 'taste disorder'/exp OR 'hearing disorder'/exp OR 'vestibular system'/exp OR 'vestibular disorder'/exp OR 'taste bud'/exp OR 'pharynx disease'/exp OR 'hypopharynx'/exp OR 'nasopharynx'/exp OR 'oropharynx'/exp OR 'adenoid'/exp OR 'tonsil disease'/exp OR 'esophagus disease'/exp OR 'ciliary dyskinesia'/exp OR 'kartagener syndrome'/exp OR 'cholesteatoma'/exp OR 'microtia'/exp OR 'otalgia'/exp OR 'herpes zoster oticus'/exp OR 'inner ear disease'/exp OR 'myringosclerosis'/exp OR 'otitis'/exp OR 'otomycosis'/exp OR 'otosclerosis'/exp OR 'ototoxicity'/exp OR 'Susac syndrome'/exp OR 'eardrum perforation'/exp OR 'laryngitis'/exp OR 'laryngocele'/exp OR 'laryngomalacia'/exp OR 'larynx stenosis'/exp OR 'supraglottitis'/exp OR 'vocal cord disorder'/exp OR 'vocal cord paralysis'/exp OR 'voice disorder'/exp OR 'choana atresia'/exp OR 'midline granuloma'/exp OR 'rhinitis'/exp OR 'rhinoscleroma'/exp OR 'dysphagia'/exp OR 'Lemierre syndrome'/exp OR 'pharyngitis'/exp OR 'mastoiditis'/exp OR 'vertigo'/exp OR 'petrositis'/exp OR 'perception deafness'/exp OR 'auditory processing disorder'/exp OR 'vestibulocochlear nerve disease'/exp OR 'dysphonia'/exp OR 'tonsillitis'/exp OR 'adenoidectomy'/exp OR 'laryngectomy'/exp OR 'laryngoplasty'/exp OR 'laryngoscopy'/exp OR 'rhinoplasty'/exp OR 'head and neck surgery'/exp OR 'auditory brain stem implant'/exp OR 'cochlea prosthesis'/exp OR 'otorhinolaryngology implant'/exp OR 'mastoidectomy'/exp OR 'middle ear ventilation'/exp OR 'myringoplasty'/exp OR 'ear surgery'/exp OR 'tympanoplasty'/exp OR 'pharyngectomy'/exp OR 'tonsillectomy'/exp OR 'tracheostomy'/exp OR 'tracheotomy'/exp OR (Otorhinolaryngolog* OR oto-laryn* OR 'head and neck' OR 'Nose Disease*' OR sino* OR nasal OR epistaxis OR Laryngeal OR Olfaction OR sinusitis OR taste OR olfactory OR smell OR phantosmia OR paraosmia OR cacosmia OR dysosmia OR anosmia OR Ageusia OR hypogeusia OR ear OR 'Vestibular System' OR nose OR 'taste buds' OR throat OR tongue OR pharynx OR pharyngeal OR larynx* OR otolaryngolog* OR Laryngolog* OR otology* OR Hypopharynx OR Nasopharynx OR Adenoid* OR Oropharyn* OR Tonsil* OR Esophageal* OR Velopharyngeal OR sinonasal OR sinus* OR paranasal OR vomeronasal OR hearing OR hear OR 'Ciliary Motility Disorder*' OR 'Kartagener Syndrome' OR Cholesteatoma OR 'Congenital Microtia' OR Earache OR oltalgia OR 'Herpes Zoster Oticus' OR 'Labyrinth Disease*' OR Myringosclerosis OR Otitis OR Otomycosis OR Otosclerosis OR Ototoxicity OR Retrocochlear OR 'Susac Syndrome' OR 'Tympanic Membrane Perforation' OR Laryngitis OR Laryngocele OR Laryngomalacia OR Laryngostenosis OR laryngotracheal OR Supraglottitis OR 'Vocal Cord Dysfunction' OR 'Vocal Cord Paralysis' OR 'Voice Disorder*' OR 'Choanal Atresia' OR 'Lethal Midline Granuloma' OR Rhinitis OR Rhinoscleroma OR 'Acoustic Neuroma' OR 'Deglutition Disorder*' OR 'Lemierre Syndrome' OR Nasopharyngeal OR Pharyngitis OR Velopharyngeal OR 'Velo-pharyngeal' OR tinnitus OR dysacusis OR paracousis OR deafness OR 'cochlear disease*' OR 'endolymphatic hydrops' OR 'Meniere disease' OR labrynthitis OR vestibulopathy OR vertigo OR ototides OR mastoiditis OR petrositis OR 'Auditory Disease*' OR 'Auditory Perceptual Disorder*' OR 'Vestibulocochlear Nerve Disease*' OR 'Vestibular Neuronitis' OR 'Vestibulocochlear Nerve Injur*' OR 'Vocal Fold Motion' OR aphonia OR dysphonia OR hoarseness OR hypopharyngeal OR nasopharyngeal OR oropharyngeal OR tonsillar OR nasopharyngitis OR retropharyngeal OR tonsillitis OR peritonsillar OR Adenoidectomy OR Laryngectomy OR Laryngoplasty OR Laryngoscopy OR Rhinoplasty OR 'Neck Dissection' OR Otologic OR 'Auditory Brain Stem Implantation' OR 'Cochlear Implant' OR 'Endolymphatic Shunt' OR 'Labyrinth Fenestration' OR Mastoidectomy OR 'Middle Ear Ventilation' OR Myringoplasty OR 'Ossicular Replacement' OR 'Stapes Surgery' OR 'Transtympanic Micropressure Treatment*' OR Tympanoplasty OR Pharyngectomy OR Pharyngostomy OR Tonsillectomy OR Tracheostomy OR Tracheotomy OR neurotization):ti,ab,kw OR (hearing OR laryng* OR 'head neck' OR otolaryng* OR oto-laryn* OR oto-rhino OR Dysphagia OR otorhino* OR acoustical OR otolog* OR rhinolog* OR Otorhinolaryngolog* OR audiolog* OR cochlear OR neurotolog* OR Vestibular OR voice OR ENT):jt OR ('ear nose' NEAR/2 throat):ti,ab,kw OR (ENT NEAR/2 disease*):ti,ab,kw OR (voice NEAR/1 (disorder* OR fatigue OR disturbance)):ti,ab,kw

**2,421,482**

**1&2 = 1459**

**OVID Medline 1.16.2024**

("contingent valuation" OR "discrete choice experiment" OR "Willingness To Pay" OR "Best-worst scaling" OR "willingness to accept" OR "stated preference*" OR "conjoint analysis" OR "conjoint survey" OR "choice model" OR "discrete choice" OR DCE OR "decision analysis" OR WTP OR "contingent valuation").ti,ab,kw.

**28,319**

exp Otorhinolaryngologic Diseases/ OR exp Otolaryngology/ OR exp Nose Diseases/ OR exp Epistaxis/ OR exp Laryngeal Diseases/ OR exp Sinusitis/ OR exp Olfaction Disorders/ OR exp Taste Disorders/ OR exp Hearing Disorders/ OR exp Vestibular System/ OR exp Vestibular Diseases/ OR exp Taste Buds/ OR exp Pharyngeal Diseases/ OR exp Hypopharynx/ OR exp Nasopharynx/ OR exp oropharynx/ OR exp Adenoids/ OR exp Palatine Tonsil/ OR exp Esophageal Diseases/ OR exp Ciliary Motility Disorders/ OR exp Kartagener Syndrome/ OR exp Cholesteatoma/ OR exp Congenital Microtia/ OR exp Earache/ OR exp Herpes Zoster Oticus/ OR exp Labyrinth Diseases/ OR exp Myringosclerosis/ OR exp Otitis/ OR exp Otomycosis/ OR exp otosclerosis/ OR exp Ototoxicity/ OR exp Susac Syndrome/ OR exp Tympanic Membrane Perforation/ OR exp Laryngitis/ OR exp Laryngocele/ OR exp Laryngomalacia/ OR exp Laryngostenosis/ OR exp Supraglottitis/ OR exp Vocal Cord Dysfunction/ OR exp Vocal Cord Paralysis/ OR exp Voice Disorders/ OR Choanal Atresia/ OR exp Granuloma, Lethal Midline/ OR exp Rhinitis/ OR exp Rhinoscleroma/ OR exp Deglutition Disorders/ OR exp Lemierre Syndrome/ OR exp Pharyngitis/ OR exp Mastoiditis/ OR exp Vertigo/ OR exp Petrositis/ OR exp Hearing Loss, Sensorineural/ OR Auditory Perceptual Disorders/ OR exp Vestibulocochlear Nerve Diseases/ OR exp Dysphonia/ OR exp Tonsillitis/ OR exp adenoidectomy/ OR exp laryngectomy/ OR exp Laryngoplasty/ OR exp Laryngoscopy/ OR exp Rhinoplasty/ OR exp Auditory Brain Stem Implants/ OR exp Cochlear Implants/ OR exp Mastoidectomy/ OR exp Middle Ear Ventilation/ OR exp myringoplasty/ OR exp Otologic Surgical Procedures/ OR exp tympanoplasty/ OR exp Pharyngectomy/ OR exp tonsillectomy/ OR exp Tracheostomy/ OR exp Tracheotomy/ OR (Otorhinolaryngolog* OR oto-laryn* OR "head and neck" OR "Nose Disease*" OR sino* OR nasal OR epistaxis OR Laryngeal OR Olfaction OR sinusitis OR taste OR olfactory OR smell OR phantosmia OR paraosmia OR cacosmia OR dysosmia OR anosmia OR Ageusia OR hypogeusia OR ear OR "Vestibular System" OR nose OR "taste buds" OR throat OR tongue OR pharynx OR pharyngeal OR larynx* OR otolaryngolog* OR Laryngolog* OR otology* OR Hypopharynx OR Nasopharynx OR Adenoid* OR Oropharyn* OR Tonsil* OR Esophageal* OR Velopharyngeal OR sinonasal OR sinus* OR paranasal OR vomeronasal OR hearing OR hear OR "Ciliary Motility Disorder*" OR "Kartagener Syndrome" OR Cholesteatoma OR "Congenital Microtia" OR Earache OR oltalgia OR "Herpes Zoster Oticus" OR "Labyrinth Disease*" OR Myringosclerosis OR Otitis OR Otomycosis OR Otosclerosis OR Ototoxicity OR Retrocochlear OR "Susac Syndrome" OR "Tympanic Membrane Perforation" OR Laryngitis OR Laryngocele OR Laryngomalacia OR Laryngostenosis OR laryngotracheal OR Supraglottitis OR "Vocal Cord Dysfunction" OR "Vocal Cord Paralysis" OR "Voice Disorder*" OR "Choanal Atresia" OR "Lethal Midline Granuloma" OR Rhinitis OR Rhinoscleroma OR "Acoustic Neuroma" OR "Deglutition Disorder*" OR "Lemierre Syndrome" OR Nasopharyngeal OR Pharyngitis OR Velopharyngeal OR "Velo-pharyngeal" OR tinnitus OR dysacusis OR paracousis OR deafness OR "cochlear disease*" OR "endolymphatic hydrops" OR "Meniere disease" OR labrynthitis OR vestibulopathy OR vertigo OR ototides OR mastoiditis OR petrositis OR "Auditory Disease*" OR "Auditory Perceptual Disorder*" OR "Vestibulocochlear Nerve Disease*" OR "Vestibular Neuronitis" OR "Vestibulocochlear Nerve Injur*" OR "Vocal Fold Motion" OR aphonia OR dysphonia OR hoarseness OR hypopharyngeal OR nasopharyngeal OR oropharyngeal OR tonsillar OR nasopharyngitis OR retropharyngeal OR tonsillitis OR peritonsillar OR Adenoidectomy OR Laryngectomy OR Laryngoplasty OR Laryngoscopy OR Rhinoplasty OR "Neck Dissection" OR Otologic OR "Auditory Brain Stem Implantation" OR "Cochlear Implant" OR "Endolymphatic Shunt" OR "Labyrinth Fenestration" OR Mastoidectomy OR "Middle Ear Ventilation" OR Myringoplasty OR "Ossicular Replacement" OR "Stapes Surgery" OR "Transtympanic Micropressure Treatment*" OR Tympanoplasty OR Pharyngectomy OR Pharyngostomy OR Tonsillectomy OR Tracheostomy OR Tracheotomy).ti,ab,kw. OR (hearing OR laryng* OR "head neck" OR otolaryng* OR oto-laryn* OR oto-rhino OR Dysphagia OR otorhino* OR acoustical OR otolog* OR rhinolog* OR Otorhinolaryngolog* OR audiolog* OR cochlear OR neurotolog* OR Vestibular OR voice OR ENT).jn OR ("ear nose" adj2 throat).ti,ab,kw. OR (ENT adj2 disease*).ti,ab,kw. OR (voice adj1 (disorder* OR fatigue OR disturbance)).ti,ab,kw.

**1,432,961**

**1&2 = 1081**

**Web of Science (Core Collection) 1.16.2024**

TS =("contingent valuation" OR "discrete choice experiment" OR "Willingness To Pay" OR "Best-worst scaling" OR "willingness to accept" OR "stated preference*" OR "conjoint analysis" OR "conjoint survey" OR "choice model" OR "discrete choice" OR DCE OR "decision analysis" OR WTP OR "contingent valuation")

**230,81924**

AND

TS=(Otorhinolaryngolog* OR oto-laryn* OR "head and neck" OR "Nose Disease*" OR sino* OR nasal OR epistaxis OR Laryngeal OR Olfaction OR sinusitis OR taste OR olfactory OR smell OR phantosmia OR paraosmia OR cacosmia OR dysosmia OR anosmia OR Ageusia OR hypogeusia OR ear OR "Vestibular System" OR nose OR "taste buds" OR throat OR tongue OR pharynx OR pharyngeal OR larynx* OR otolaryngolog* OR Laryngolog* OR otology* OR Hypopharynx OR Nasopharynx OR Adenoid* OR Oropharyn* OR Tonsil* OR Esophageal* OR Velopharyngeal OR sinonasal OR sinus* OR paranasal OR vomeronasal OR hearing OR hear OR "Ciliary Motility Disorder*" OR "Kartagener Syndrome" OR Cholesteatoma OR "Congenital Microtia" OR Earache OR oltalgia OR "Herpes Zoster Oticus" OR "Labyrinth Disease*" OR Myringosclerosis OR Otitis OR Otomycosis OR Otosclerosis OR Ototoxicity OR Retrocochlear OR "Susac Syndrome" OR "Tympanic Membrane Perforation" OR Laryngitis OR Laryngocele OR Laryngomalacia OR Laryngostenosis OR laryngotracheal OR Supraglottitis OR "Vocal Cord Dysfunction" OR "Vocal Cord Paralysis" OR "Voice Disorder*" OR "Choanal Atresia" OR "Lethal Midline Granuloma" OR Rhinitis OR Rhinoscleroma OR "Acoustic Neuroma" OR "Deglutition Disorder*" OR "Lemierre Syndrome" OR Nasopharyngeal OR Pharyngitis OR Velopharyngeal OR "Velo-pharyngeal" OR tinnitus OR dysacusis OR paracousis OR deafness OR "cochlear disease*" OR "endolymphatic hydrops" OR "Meniere disease" OR labrynthitis OR vestibulopathy OR vertigo OR ototides OR mastoiditis OR petrositis OR "Auditory Disease*" OR "Auditory Perceptual Disorder*" OR "Vestibulocochlear Nerve Disease*" OR "Vestibular Neuronitis" OR "Vestibulocochlear Nerve Injur*" OR "Vocal Fold Motion" OR aphonia OR dysphonia OR hoarseness OR hypopharyngeal OR nasopharyngeal OR oropharyngeal OR tonsillar OR nasopharyngitis OR retropharyngeal OR tonsillitis OR peritonsillar OR Adenoidectomy OR Laryngectomy OR Laryngoplasty OR Laryngoscopy OR Rhinoplasty OR "Neck Dissection" OR Otologic OR "Auditory Brain Stem Implantation" OR "Cochlear Implant" OR "Endolymphatic Shunt" OR "Labyrinth Fenestration" OR Mastoidectomy OR "Middle Ear Ventilation" OR Myringoplasty OR "Ossicular Replacement" OR "Stapes Surgery" OR "Transtympanic Micropressure Treatment*" OR Tympanoplasty OR Pharyngectomy OR Pharyngostomy OR Tonsillectomy OR Tracheostomy OR Tracheotomy) OR SO=(hearing OR laryng* OR "head neck" OR otolaryng* OR oto-laryn* OR oto-rhino OR Dysphagia OR otorhino* OR acoustical OR otolog* OR rhinolog* OR Otorhinolaryngolog* OR audiolog* OR cochlear OR neurotolog* OR Vestibular OR voice OR ENT)

TS=("ear nose" NEAR/2 throat) OR (ENT NEAR/2 disease*) OR (voice NEAR/1 (disorder* OR fatigue OR disturbance))

**1,506,635**

**1&2 = 2,235**
