# Supplementary material for: Stated Preference Research in Otolaryngology: A Scoping Review
Source: OTO Open. 2025 Jun 12;9(2):e70140. doi: 10.1002/oto2.70140 (PMC12160337; doi:10.1002/oto2.70140)
Supplement: Supplementary file 2 — Supplemental Table S1. [file OTO2-9-e70140-s002.docx]

**Supplemental Table 1.** Included studies with relevant attributes. (WTP=willingness to pay; CV=contingent valuation; DCE=discrete choice experiment; CA=conjoint analysis; BWS=best-worst scaling; NA=not applicable. An asterisk * indicates no funding information was reported in manuscript.)

| **Author /Year** | **Country** | **Study objective** | **Sub-specialty** | **Population** | **n** | **Study type** | **Preference measured** | **Development method** | **WTP method** | **# attributes / # choice sets** | **Probability sampling** | **Findings** |
| --- | --- | --- | --- | --- | --- | --- | --- | --- | --- | --- | --- | --- |
| Barbe 2019 | Germany | To determine the efficacy of a new symptom relieving mouth gel vs. a widely used control gel on xerostomic burden | General otolaryngology | Adults with moderate xerostomia | 32 | WTP/CV | Treatment (medication) | Not specified | Bidding game | NA | Yes | No significant differences in WTP between test and control gel |
| Bogelund / 2022 | Denmark, Spain | To understand preference for allergy immunotherapy, sublingual vs subcutaneous | Rhinology; Allergy | Adults and caregivers of children with allergic rhinitis | 1190 | DCE | Treatment (medication) | Not specified | NA | 3 / 8 | No | Respondents prefer sublingual therapy |
| Braun 2022 | Germany, USA | To evaluate treatment preferences and WTP among patients with and without previous experience of OSA therapies | Sleep | Adults with OSA presenting for overnight polysomnography | 241 | DCE; WTP/CV | Treatment (medication); Treatment (procedure); Treatment (device) | Literature review; Pilot study | Within DCE/CA | 7 / 4 | No | Positive airway pressure is most preferred |
| Bridges  2012 | USA | To directly compare Likert scales and conjoint analysis in identifying important attributes associated with hearing aids among those with hearing loss | Otology / Audiology | Adults with sensorineural hearing loss beyond 30 decibel speech reception threshold | 56 | CA; WTP/CV | Treatment (device) | Expert opinion; Interview ; Pilot study | Within DCE/CA | 7 / 8 | No | Improved performance in noisy settings is the most valued attribute of hearing aids |
| Chen 2019 | USA | To determine the monetary value of changes in attractiveness achieved through rhinoplasty, as perceived by society | Facial plastics | Casual observers online | 228 | WTP/CV | Treatment (procedure) | Not specified | VAS | NA | No | Change in attractiveness after rhinoplasty has a monetary value commensurate with the actual average cost of rhinoplasty* |
| Chenevier 2005 | Canada | To assess parental WTP for a shorter course of antibacterial treatment versus conventional therapy for acute otitis | Pediatrics | Parents of children treated for acute otitis media | 562 | WTP/CV | Treatment (medication) | Literature review | Open ended; Bidding game | NA | No | Parents of children with AOM were willing to pay more for their child to benefit from a shorter duration of antibacterial treatment than for a standard course |
| Cheung  2010 | USA | To demonstrate methodology and feasibility of adapting conjoint analysis for mapping clinical outcomes expectations to treatment decisions in vestibular schwannoma management | Otology / Audiology | Vestibular schwannoma patients, surgeons, and controls | 256 | CA | Treatment (procedure) | Expert opinion; Literature review | NA | 4 / 9 | No | Permanent deafness was less important to tumor surgeons, and temporary facial weakness was more important to tumor surgeons and observation patients |
| Chisolm  2001 | USA | To assess value associated with hearing aid benefit | Otology / Audiology | VA hearing aid patients | 103 | WTP/CV | Treatment (device) | Not specified | Open ended | NA | No | WTP was $981.71 on average, and participants willing to may most for benefit of understanding speech above background noise* |
| Damm  2016 | Germany | To elicit patient preferences to identify the allergy immunotherapy administration mode preferred by patients | Rhinology; Allergy | Adults with moderate to severe allergic rhinitis, no previous immunotherapy | 239 | DCE | Treatment (medication) | Expert opinion; Literature review | NA | 5 / 8 | No | Patients strongly prefer fewer visits, lower risk of shock, and avoidance of tongue swelling |
| Ding  2022 | USA | To investigate public perceptions of radiofrequency ablation, including WTP | Head and neck oncology | General adult population | 830 | WTP/CV | Treatment (procedure) | Not specified | VAS | NA | No | General population respondents are willing to pay less for radiofrequency ablation vs. surgery. |
| Dusseldorp  2021 | USA | To determine preferences between surgical options for facial reanimation | Facial plastics | Adult community volunteers | 241 | DCE | Treatment (procedure) | Expert opinion; Literature review; Interview ; Pilot study | NA | 5 / 12 | No | Smile reanimation by cross-facial vs. trigeminal neurotization is favored by most participants |
| Engineer 2013 | USA | To determine WTP for implantable devices for tinnitus | Otology / Audiology | Respondents on tinnitus email distribution list | 439 | WTP/CV | Treatment (procedure); Treatment (device) | Not specified | Payment card | NA | No | Approximately 3/4 of respondents would have a device implanted if it reduced tinnitus by half |
| Etzkorn  2018 | USA | To evaluate preferences for surgical treatment of facial melanoma | Head and neck oncology | Adults presenting to academic medical center with melanoma | 158 | CA | Treatment (procedure) | Not specified | Within DCE/CA | 6 / 12 | Yes | Patients prefer surgical treatment options that minimize risk for local recurrence |
| Fifer 2023 | Australia, India, UK | To understand importance of treatment attributes of nasal steroids and antihistamines | Rhinology; Allergy | Adults with moderate to severe AR in online panel | 426 | BWS | Treatment (medication) | Not specified | NA | 11 / 11 | No | Satisfaction with treatment for allergic rhinitis is associated with the sensory attributes of the medication |
| Fischman 2021 | USA | To quantitatively examine preferences among patients choosing a laryngologist | Laryngology | New patients presenting to laryngology clinic | 87 | BWS | Treatment (procedure) | Expert opinion; Literature review; Interview ; Pilot study | NA | 13 / 12 | No | Nonclinical factors were less important to patients than clinical factors, and laryngology-specific training was paramount |
| Fitzpatrick 2018 | Canada | To examine the preferences for characteristics associated with intervention services | Otology / Audiology | Parents of young children with permanent mild bilateral and unilateral hearing loss | 51 | CA | Treatment (procedure) | Expert opinion; Literature review; Interview | NA | 4 / 8 | No | Patients indicated a clear preference for speech-language support compared with support for amplification use |
| Fitzpatrick 2007 | Canada | To examine preferences of parents regarding interventional services for hearing impairment | Otology / Audiology | Parents of children enrolled in interventional programs with a focus on oral communication | 48 | CA | Treatment (procedure) | Literature review; Interview | NA | 5 / 8 | No | Parents preferred clinic-based services less frequently to more frequent home-based |
| Grose  2021 | Canada | To explore patient perspectives regarding out of pocket payment for tonsillectomy and septoplasty | General otolaryngology | Patients scheduled to undergo septoplasty or tonsillectomy | 71 | WTP/CV | Treatment (procedure) | Expert opinion | Dichotomous choice | NA | No | 21% of patients were willing to pay out-of-pocket for a surgery |
| Grutters  2008 | The Netherlands | To compare willingness to accept and WTP in a discrete choice experiment on hearing aid provision | Otology / Audiology | Persons with hearing complaints visiting a hearing aid dispenser | 291 | DCE; WTP/CV | Treatment (device) | Expert opinion; Interview ; Pilot study | Within DCE/CA | 5 / 17 | No | Willingness to accept a discount exceeds WTP for hearing aids |
| Grutters  2008 | The Netherlands | To understand the trade-offs between different elements of hearing aid provision | Otology / Audiology | Hearing impaired adults | 146 | DCE | Treatment (device) | Expert opinion; Pilot study | NA | 5 / 16 | No | Hearing-impaired persons are receptive to transferring elements of hearing aid provision from the medical sector to private dispensers |
| Grutters  2009 | The Netherlands | To evaluate the validity of two WTP elicitation formats in the context of hearing loss | Otology / Audiology | Adult hearing aid users | 108 | WTP/CV | Treatment (device) | Literature review | Open ended; Payment card | NA | No | WTP results elicited with the payment scale and open-ended question werenot statistically significantly different |
| Keith  2000 | Canada | To determine the WTP for intranasal budesonide | Rhinology; Allergy | Symptomatic patients with allergic rhinitis | 242 | WTP/CV | Treatment (medication) | Other | Open ended | NA | Yes | Intranasal budesonide is cost-beneficial in the treatment of allergic rhinitis* |
| Kumar  2020 | USA | To analyze and compare the value of rhytidectomy in an established market and an emerging market | Facial plastics | General population in USA and India | 236 | WTP/CV | Treatment (procedure) | Not specified | VAS | NA | No | WTP for rhytidectomy is nonlinear and exponentially increases with improved appearance* |
| Kumar  2020 | USA | To measure and quantify the health state utility and WTP for cosmetic rhinoplasty | Facial plastics | Casual observers from general population | 167 | WTP/CV | Treatment (procedure) | Not specified | Open ended | NA | No | WTP for cosmetic rhinoplasty is approximately $15k |
| Mahadevia  2004 | USA | To evaluate patient preferences for intranasal steroid sensory attributes | Rhinology; Allergy | Adult patients with allergic rhinitis across the USA | 120 | CA | Treatment (medication) | Not specified | NA | 6 / 15 | No | Sensory attributes are negatively associated with preference |
| Mahadevia 2006 | USA | To determine WTP for presence or lack of sensory attributes of intranasal steroids | Rhinology; Allergy | Adult patients with allergic rhinitis and history of intranasal steroid use | 120 | DCE | Treatment (medication) | Pilot study | Within DCE/CA | 7 / 8 | No | Patients were most willing to pay for lack of taste or aftertaste. |
| Martin 2016 | Germany | To assess the preferences of patients with basal cell carcinoma for outcome and process attributes | Head and neck oncology | Adult patients with suspected or confirmed basal cell carcinoma | 124 | CA | Treatment (procedure) | Expert opinion; Literature review | NA | 11 / 12 | No | Participants with basal cell carcinoma of the head or neck were particularly interested in cosmetic outcome* |
| Meister 2002 | Germany | To determine the importance of hearing-aid attributes | Otology / Audiology | Experienced hearing aid users | 200 | CA | Treatment (device) | Pilot study | NA | 6 / 8 | Yes | Older and younger hearing-aid users differ only slightly regarding fundamental aspects of amplification |
| Meister 2002 | Germany | To determine the importance of hearing aid attributes; to elicit measures of satisfaction | Otology / Audiology | Experienced hearing aid users | 175 | CA | Treatment (device) | Pilot study | NA | 6 / 8 | Yes | Speech in quiet and speech in noise were the two most important features identified by participants |
| Meister 2001 | Germany | To measure the contributions of different hearing aid attributes involved in preference | Otology / Audiology | Experienced hearing aid wearers | 93 | CA | Treatment (device) | Pilot study | Within DCE/CA; NA | 6 / 8 | Yes | Elder and female subjects attached greater importance of the attribute handling whereas younger patients judged speech in noise more important |
| Meregaglia 2017 | Italy, United Kingdom | To assess patient preferences for direct aspects of follow up care characteristics | Head and neck oncology | Adult outpatient head and neck cancer patients | 143 | BWS | Diagnosis | Literature review; Interview | NA | 4 / 9 | No | A preference for a hospital-based surveillance program after treatment with decreasing frequency over time was shown |
| Naunheim 2017 | USA | To assess WTP in the context of an ophthalmology and otolaryngology emergency department | General otolaryngology | Ophthalmology and otolaryngology adult emergency room patients | 327 | WTP/CV | Treatment (procedure) | Literature review; Interview ; Pilot study | Payment card | NA | No | Patients with eye and ear, nose, and throat complaints place a mean explicit value on specialty emergency services of $340 per visit |
| Naunheim 2015 | USA | To provide a cost-benefit analysis of otolaryngology-specific emergency room care | General otolaryngology | Adult patients presenting to an otolaryngology emergency room | 199 | WTP/CV | Treatment (procedure) | Literature review; Interview ; Pilot study | Payment card; Dichotomous choice | NA | No | Dedicated otolaryngology ER services are valued by patients for acute otolaryngologic problems and demonstrable monetary |
| Naunheim 2017 | USA | To understand how attributes of an ENT clinic (including wait time, physician characteristics, visit utilization, cost) are assessed by patients | General otolaryngology | Adults from general population | 161 | DCE | Treatment (procedure) | Literature review; Interview ; Pilot study | NA | 5 / 14 | No | Patients are willing to pay extra for timely appointments, physicians with experience, and a thorough physical examination. |
| Naunheim 2017 | USA | To assess treatment preferences for airway stenosis relative to endoscopic and open surgery | Laryngology | Adult members of general population | 162 | DCE | Treatment (procedure) | Expert opinion; Literature review; Interview | NA | 5 / 14 | No | Most participants preferred voice-sparing, low-risk procedures as treatment for subglottic stenosis, consistent with an endoscopic approach, even if multiple procedures were required. |
| Nickel 2018 | Australia | To determine which factors influence treatment preferences and tradeoffs for patients with papillary thyroid carcinoma, and to understand how terminology influences preferences | Head and neck oncology | General public online research panel | 2054 | DCE | Treatment (procedure) | Literature review; Interview ; Pilot study | NA | 8 / 12 | Yes | Participants were willing to accept a higher number of extra patients experiencing adverse effects to avoid a thyroid cancer death when the condition was described as a cancer compared with a lesion |
| Osmond 1995 | Canada | To determine the most cost efficient method of pediatric facial laceration repair | Facial plastics; Pediatrics | Parents of children in emergency room without facial lacerations | 30 | WTP/CV | Treatment (procedure) | Not specified | Open ended | NA | No | Tissue adhesive is the preferred method of closure of pediatric facial lacerations, with a higher WTP by parents* |
| Petersen 2010 | Denmark | To elicit WTP for allergen-specific subcutaneous injection immunotherapy in with allergic rhinoconjunctivitis, and to investigate how patients self-select to treatment | Rhinology; Allergy | General population with rhinoconjunctivitis symptoms who had not received any immunotherapy treatment | 317 | WTP/CV | Treatment (medication) | Not specified | Open ended; Dichotomous choice | NA | Yes | A minority of patients were willing to pay a positive amount for immunotherapy |
| Phillips 2020 | Canada | To discern the value of benefit risk information related to systemic therapy in head and neck, breast and colorectal cancer | Head and neck oncology | Patients with head and neck cancer who were contemplating, receiving or had previously received systemic therapy | 293 | DCE | Treatment (medication) | Expert opinion; Pilot study | NA | 3 /10 | No | Benefit was the most important decision attribute regardless of treatment intent, followed by risk of hospitalization, then risk of ED visit |
| Rademaker 2021 | The Netherlands | To study the preference of the tinnitus patient for different outcome measures in tinnitus therapy | Otology / Audiology | Adults seeking care for tinnitus | 127 | DCE | Heath state | Literature review; Focus group | NA | 4 / 9 | No | Tinnitus loudness was considered the most important outcome measure, with latent class analysis demonstrating distinct preference phenotypes |
| Rallapalli 2002 | USA | To determine how multiple types of signal processing activated together influence listener's preferences | Otology / Audiology | Adults with mild to moderately severe sensorineural hearing loss | 36 | CA | Treatment (device) | Other | NA | 3 / 64 | No | Signal processing characteristics significantly influence preference, but effect sizes are small and may not result in substantial change in clinical outcomes |
| Reardon 1990 | USA | To determine the value of antihistamine drug products to allergic rhinitis sufferers with a history of antihistamine use | Rhinology; Allergy | University employees with rhinitis symptoms and antihistamine use | 143 | CA; WTP/CV | Treatment (medication) | Expert opinion; Literature review; Focus group | Open ended | 7 / 16 | Yes | Using CV and WTP data, distinct patient clusters can be shown* |
| Riethmuller 2008 | Switzerland | To determine the monetary value of a night of undisturbed sleep | Sleep | Patients with obstructive sleep apnea syndrome controlled by nasal continuous positive airway pressure | 67 | WTP/CV | Heath state | Interview | Open ended | NA | No | The monetary value of 70 CHF for a night of undisturbed sleep was greater than the cost of CPAP therapy (5) or daily rent (40-50) |
| Sach 2004 | United Kingdom | To measure the value parents place on having a cochlear implantation program | Otology; Pediatrics | Parents of children involved with a cochlear implant program | 216 | WTP/CV | Treatment (procedure) | Interview ; Focus group; Other | Bidding game; Dichotomous choice | NA | No | Parents were willing to pay a mean of 127 GBP monthly to fund a cochlear implantation program. 99% of parents choose the implant over spending this money in a different way |
| Schafer 2002 | Germany | To determine usage of alternative medication for allergies, including WTP | Allergy | Adults >25 years old with hay fever, asthma, eczema, or food hypersensitivity | 351 | WTP/CV | Treatment (medication) | Not specified | Open ended | NA | Yes | Alternatives medicines are widely used, and patients are willing to pay high substantial amounts for perceived benefit |
| Schulz 2023 | Germany | To examine preferences about the long term care needs for cochlear implants | Otology / Audiology | Adults with cochlear implant | 92 | DCE | Treatment (device) | Literature review; Interview ; Focus group | NA | 6 / 36 | No | Technological compatibility of the implant with newer implant models, accessories or devices from other manufacturers was highly valued |
| Sorum 1999 | USA | To demonstrate decision makers WTP to avoid undesirable events related to otitis media | Otology; Pediatrics | Parents presenting with children for sick or well visits in a suburban medicine and pediatrics office | 219 | WTP/CV | Heath state; Treatment (medication); Treatment (procedure) | Not specified | Open ended | NA | No | Permanent hearing loss was most negatively valued; willingess to pay outweighed costs when treating with antibiotics* |
| Sukpanich 2021 | USA, Thailand | To evaluate the influence of cosmetic concerns and other factors on patients' decision-making processes when choosing among different thyroidectomy approaches | Head and neck oncology | Adult patients seen in endocrine surgery clinic at a high volume urban academic medical center | 109 | DCE; WTP/CV | Treatment (procedure) | Expert opinion; Literature review; Focus group | Within DCE/CA | 5 / 9 | No | The risk of having RLN and/or mental nerve injury, travel distance, and cost were more important than cosmesis, although younger patients may pay more to avoid a scar |
| Tankersley 2021 | USA, Denmark | To investigate patient preference for sublingual versus subcutaneous terapies for allergic rhinitis | Rhinology; Pediatrics; Allergy | Adults with allergic rhinitis, and caregivers of children with allergic rhinitis | 1389 | DCE; WTP/CV | Treatment (medication) | Not specified (DCE); Other (WTP) | Payment card | 3 / 8 | No | Both adults and caregivers had a significant preference for SLIT-tablets compared with both weekly and monthly injections; the younger the child, the more risk-averse the caregiver |
| Tarasiuk 2003 | Israel | To analyze WTP for polysomnography among parents of children with obstructive sleep apnea syndrome, with cost-benefit analysis | Pediatrics; Sleep | Consecutively recruited parents of children with OSA | 252 | WTP/CV | Diagnosis | Not specified | Bidding game | NA | No | Average WTP of $762; parents of older children were less inclined to pay compared to younger children |
| Tyler 2012 | USA | To understand the range of perceptions about tinnitus treatment and assess WTP for treatment and cure | Otology / Audiology | Audience members of the Austrailian Tinnitus Society Meeting in 2008 | 197 | WTP/CV | Treatment (medication); Treatment (procedure); Treatment (device) | Not specified | Payment card | NA | No | To reduce tinnitus completely, participants most commonly selected to pay at least $5000; one fifth were willing to pay as much as $25,000* |
| Upton 2023 | USA | To understand opinions about divergent treatment options for advanced laryngeal cancer | Laryngology; Head and neck oncology | Healthy adult volunteers | 301 | CA | Heath state | Expert opinion | NA | 7 / 15 | No | General public opinion ranked lifespan, voicing, and swallowing aspects as similarly important, and all were ranked more important than probability of cure |
| van der Pol 2010 | United Kingdom | To assess the costs and benefits of a tele-endoscopy service in Scotland | General otolaryngology | Adult members of the general public in Scotland | 90 | DCE; WTP/CV | Diagnosis | Not specified | Payment card | 4 / 16 | Yes | Tele-endoscopy clinics in Shetland are efficient, cost-effective at scale; patients have high WTP for reduced travel time |
| Wilson 2023 | United Kingdom | To establish the clinical effectiveness and cost-effectiveness of tonsillectomy for adult tonsillitis; in particular to assess WTP to avoid a day of sore throat | General otolaryngology | Adult patients with recurrent sore throats | 413 | WTP/CV | Heath state | Not specified | Payment card | NA | Yes | The mean WTP of 43 GBP is greater than the cost of prevention |
| Yueh 2001 | USA | T compare the effectiveness of an assistive listening device, hearing aids, microphones against no amplification; to assess WTP for amplification devices | Otology / Audiology | Patients 50+ seeking diagnostic visit or hearing aid evaluation | 60 | WTP/CV | Treatment (device) | Not specified | Open ended | NA | Yes | Patients would be willing to pay more for programmable vs. standard hearing aids |
| Zhang 2021 | USA | To understand how general perceptions about CI contribute to barriers causing this low utilization rate | Otology / Audiology | Online adult US participants | 615 | WTP/CV | Treatment (procedure); Treatment (device) | Not specified | Payment card | NA | No | Respondents identified issues with insurance and fear of undergoing surgery as barriers preventing eligible patients from receiving CI* |
| Zhu 2020 | China | To elicit the preferences for hearing aid attributes among rural Chinese adults with moderate or greater hearing loss | Otology / Audiology | Adults in rural Shandong province | 125 | DCE; WTP/CV | Treatment (device) | Literature review; Interview | Within DCE/CA | 8 / 8 | No | Effectiveness in noisy settings was valued above all other attributes, with willingness to pay $571 additional for this feature on average |
